# Epidemiological, Clinical, Radiological Characteristics and Outcomes of Medical Staff with COVID-19 in Wuhan, China: Analysis of 101 Cases

**DOI:** 10.7150/ijms.54257

**Published:** 2021-01-29

**Authors:** Jie Liu, Liu Ouyang, Dan Yang, Xiaoyu Han, Yukun Cao, Osamah Alwalid, Hanping Wu, Heshui Shi, Fan Yang, Chuansheng Zheng

**Affiliations:** 1 Department of Radiology, Union Hospital, Tongji Medical College, Huazhong University of Science and Technology, Wuhan 430022, China.; 2 Hubei Province Key Laboratory of Molecular Imaging, Wuhan 430022, China.; 3 Department of Orthopaedics, Union Hospital, Tongji Medical College, Huazhong University of Science and Technology, Wuhan 430022, China.; 4 Department of Respiratory and Critical Care Medicine, Union Hospital, Tongji Medical College, Huazhong University of Science and Technology, Wuhan 430022, China.; 5 Department of Radiology, Michigan Medicine, University of Michigan, Michigan, The United States of America.

**Keywords:** COVID-19, Medical staff, Infectious disease, Epidemiology, Clinical features, Computerized tomography.

## Abstract

**Objectives:** As of 11 Feb 2020, a total of 1,716 medical staff infected with laboratory-confirmed the severe acute respiratory syndrome coronavirus 2 (SARS-Cov-2) in China had been reported. The predominant cause of the infection among medical staff remains unclear. We sought to explore the epidemiological, clinical characteristics and prognosis of infected medical staff.

**Methods:** Medical staff who infected with SARS-Cov-2 and admitted to Union Hospital, Wuhan between 16 Jan to 25 Feb, 2020 were included in this single-centered, retrospective study. Data were compared by occupation and analyzed with the Kaplan-Meier and Cox regression methods.

**Results:** A total of 101 medical staff (32 males and 69 females; median age: 33) were included in this study and 74.3% were nurses. A small proportion of the cohort had contact with specimens (3%) as well as patients infected with SARS-Cov-2 in fever clinics (15%) and isolation wards (3%). 80% of medical staff showed abnormal IL-6 levels and 33% had lymphocytopenia. Chest CT mainly manifested as bilateral (62%), septal/subpleural (77%) and groundglass opacities (48%). The major differences between doctors and nurses manifested in laboratory indicators. As of the last observed date, no patient was transferred to intensive care unit or died. Fever (HR=0.57; 95% CI 0.36-0.90) and IL-6 levels greater than 2.9 pg/ml (HR=0.50; 95% CI 0.30-0.86) were unfavorable factors for discharge.

**Conclusions:** Our findings suggested that the infection of medical staff mainly occurred at the early stages of SARS-CoV-2 epidemic in Wuhan, and only a small proportion of infection had an exact mode. Meanwhile, medical staff infected with COVID-19 have relatively milder symptoms and favorable clinical course than ordinary patients, which may be partly due to their medical expertise, younger age and less underlying diseases. The potential risk factors of fever and IL-6 levels greater than 2.9 pg/ml could help to identify medical staff with poor prognosis at an early stage.

## Introduction

In Dec 2019, a group of novel atypical pneumonia patients with uncertain etiology but mostly linked to the Huanan Seafood Wholesale Market emerged in Wuhan, China 1. A later confirmed pathogen of this previously unknown pneumonia was described as a novel coronavirus, currently named as severe acute respiratory syndrome coronavirus 2 (SARS-Cov-2), was ascertained by unbiased sequencing analysis of lower respiratory tract samples from early cases on 7 Jan 2020, following which the protocol of real-time reverse-transcriptase polymerase chain reaction (RT-PCR) assay for this novel coronavirus had also been developed 2, 3. By 5 October 2020, coronavirus disease 2019 (COVID-19) due to the SARS-Cov-2 has caused 34,804,348 cases laboratory confirmed cases and 1,030,738 deaths among them globally 4. Sufficient evidence indicated that the COVID-19 clustered within close-contact human groups, such as family and hospital settings 5-7.

Information pointing to the epidemiology and clinical features of general confirmed cases has been accumulating 1, 8, 9. Meanwhile, a finding from a national wide descriptive report drew a huge amount of attention, which declared that the total number of confirmed novel coronavirus-infected medical staff was as high 1,716 as of 11 February 2020, with a peak incidence occurring on Jan 28, 2020 10. Among them, 63% (1080) were in Wuhan 10. A cluster of 14 medical staff infected with COVID-19 from department of neurosurgery in Wuhan Union Hospital were initially reported 11. The previous studies enrolling 14, 80 and 54 hospitalized frontline medical workers infected with COVID-19 respectively, provided an insight into epidemiological and clinical characteristics of these patients 11-13. Nonetheless, information regarding the clinical outcomes and potential risk factors of medical staff confirmed with COVID-19 remains to be investigated.

At present, there are increased attentions paid to protecting medical staff from infection, and medical staff are regarded as every country's most valuable resource to fight against the COVID-19 outbreak 14. People paid tribute to healthcare workers for their efforts during the outbreak of COVID-19 15. Based on a group of medical staff confirmed with COVID-19 who were admitted to Union Hospital, Wuhan, this retrospective study aimed to reveal some epidemiological and clinical findings, and identify potential risk factors of extended hospitalization. We hope the findings will provide an insight into the prevention and treatment of this novel coronavirus for the global community.

## Patients and Methods

This retrospective study was approved by the Ethics of Committees of Union Hospital, Tongji Medical College, Huazhong University of Science and Technology. Written informed consent was waived due to the rapid emergence of this infectious disease.

### Study design and participants

This is a single-centered, retrospective study on a group of SARS-CoV-2 infected medical staff at Wuhan Union Hospital, one of the hospitals treating patients with COVID-19 at the earliest time. Diagnosis of cases with SARS-Cov-2 infection conforms to the WHO interim guidance 16. Details regarding laboratory confirmation protocol for SARS-CoV-2 were described by previous studies 1, 9. Throat-swab specimens were screened for SARS-CoV-2 and other respiratory viruses (influenza, respiratory syncytial virus, etc.) by real-time RT-PCR assays. This study only considered medical staff that are in service. A total 101 medical staff, which were confirmed by SARS-CoV-2 real-time RT-PCR test on respiratory secretions collected by throat swab and undergone serial chest CT scans following their admission to isolation wards of Union Hospital between 16 Jan and 25 Feb, 2020 were included.

### Data collection

The epidemiological data, medical and nursing records, laboratory examinations, chest computed tomography (CT) of all patients were reviewed and abstracted with concerted efforts of experienced clinicians. Data were collected at the time of symptoms onset, presentation for medical advice and in-patient admission. The clinicians who had experience of treating patients with confirmed SARS-Cov-2 infection reviewed and collected the medical records of patients, and preliminarily collated the data. The clinical data were extracted through a standardized form for case report as previously described 17. Epidemiological data, including exposure histories before symptoms onset (whether there is a history of exposure to the Huanan Seafood Wholesale Market, or wildlife), and close contact with laboratory-confirmed or suspected cases of COVID-19 in work environment (fever clinics, or isolation wards) and sample collection sites (with pharyngeal swab, blood, sputum specimens, etc.), or close contact with family members with COVID-19 were collected. Also, information about preventive medication among medical staff was collected.

We have also collected the data on demographics, clinical manifestations, laboratory examinations and radiological studies. These included age, sex, occupation (doctor, or nurse), body mass index (BMI ≥24, or <24 kg/m^2^), current smoking status (yes, or no), disease severity (non-severe, or severe), date of symptom onset, diagnosis and hospital admission, symptoms before hospital admission (fever, cough, fatigue, sore throat, myalgia, sputum production, difficulty breathing or chest tightness, chill, loss of appetite, diarrhea, and chest pain), coexisting conditions (e.g. hypertension, diabetes, etc.), laboratory testing indicators on admission (leucocyte count, lymphocyte count, platelet count, D-dimer, creatinine, creatine kinase, lactose dehydrogenase, alanine aminotransferase, aspartate aminotransferase, hemoglobin, ferritin, C-reactive protein, Amyloid A, total bilirubin, procalcitonin, erythrocyte sedimentation rate, interleukin-6 (IL-6) and lymphocyte subsets, etc.), radiologic assessments of chest CT (lung involvement, lung lobe involvement, predominant CT changes, predominant distribution of opacities, etc.), treatment measures (antibiotics agents, antiviral agents, traditional Chinese medicine, immune globulin, thymosin, corticosteroids and oxygen therapy), and complications (e.g. pneumonia, acute respiratory distress syndrome, acute cardiac injury, acute kidney injury, shock, etc.). All CT images were analyzed by two radiologists (J.L. and F.Y., who had 5 and 21 years of experience in thoracic radiology, respectively) utilizing the institutional digital database system without access to clinical and laboratory findings. Images were reviewed independently, and final decisions were reached by discussion and consensus. We estimated the time interval from symptom onset to diagnosis and admission with maximum information available - that is, all the exact date of initial symptoms provided by the patients. Then the aggregated data was sent to data analysis group. Prior to statistical analysis, the aggregated data were cross - checked by group members to guarantee the correctness and completeness of data.

### Outcomes

The clinical outcomes and prognosis were continuously observed up to Mar 20, 2020. The primary end point was discharge, needed to meet the following three conditions 18: (1) body temperature return to normal for more than 3 days and respiratory symptoms improvement; (2) improvement of lung involvement demonstrated by chest CT; (3) two consecutive negative RT-RCR tests, with sampling interval of more than 1 day. Secondary outcomes consisted of hospital discharge rate.

### Statistical analysis

This study devoted to report epidemiological, clinical characteristics and prognosis of medical staff confirmed with COVID-19. We estimated the distributions of durations from symptoms onset to diagnosis, symptoms onset to admission, and diagnosis to admission, respectively. Kaplan-Meier method was applied to estimate the change in hospital discharge rate. The proportional hazard Cox regression model was utilized to ascertain potential factors associated with discharge. Univariate models with a single variable once at a time were first fitted. The statistically significant risk factors as well as age and sex were, then, would be considered and selected into a final multivariate Cox regression model. The hazards ratios (HRs) along with the 95% confidence intervals (95% CIs) were calculated.

Statistical tests were two-sided with significance set at *α* less than 0.05. We performed all data analyses by R software version 3.6.2 (R Foundation for Statistical Computing).

## Results

### Epidemiological characteristics

During the study period, epidemiological and clinical data were collected on 101 medical staff with laboratory-confirmed SARS-Cov-2 infection from Wuhan Union Hospital, of whom 99 (98%) provided an exact date of symptom onset and only 6 cases (6%) were severe. The patients aged between 23 and 63 years old, and median age was 33 years (IQR 30-41 years) (Table 1). More than half of the cohort were female (68%) and nurse (74%). There were 18 (18%) cases with a large BMI (BMI ≥ 24 kg/m^2^), and 4 (4%) were current smokers.

Among the 101 medical staff recruited, no one had an exposure to Huanan seafood wholesale market or wildlife, while 6 (6%) medical staff had family members confirmed with SARS-CoV-2 infection. During patient care, 15 (15%), 3 (3%) and 3 (3%) cases had direct contact with patients in fever clinics, isolation wards and specimens collected from patients, respectively. 10 (10%) of 101 medical staff have used preventive medications. No major differences of exposure history and preventive medications were observed between the two occupational types, except for the contact with specimens were more common in doctors (*P*-value<0.05).

**Figure 1:**
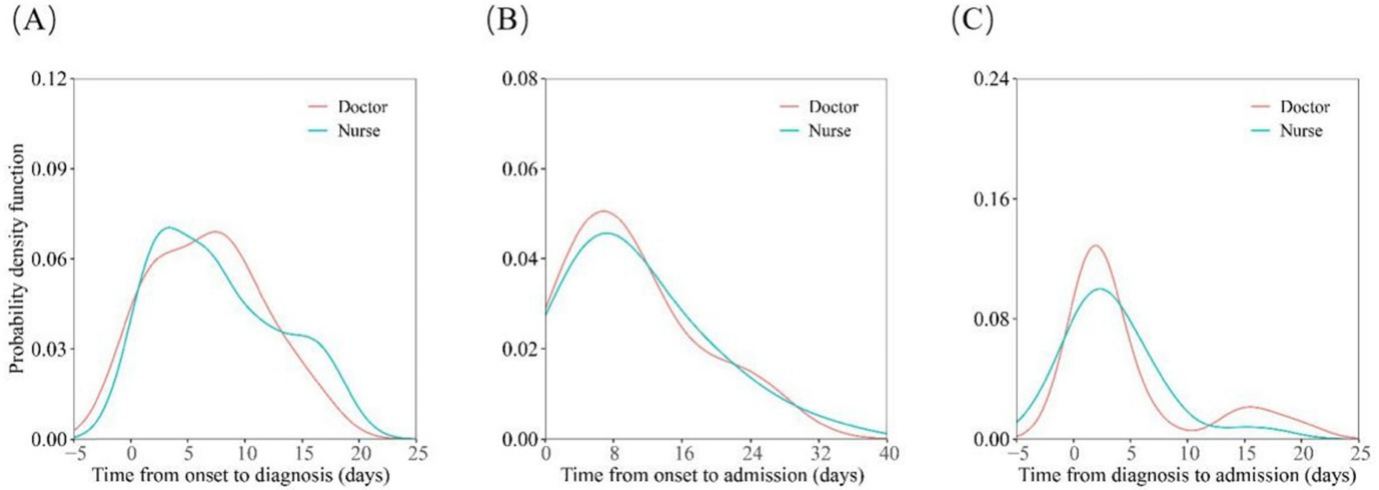
** Distributions of date of diagnosis, onset-to-diagnosis duration, onset-to-admission duration and diagnosis-to-admission duration.** (A) Estimates of onset-to-diagnosis distribution stratified by occupation. (B) Estimates of onset-to-admission distribution stratified by occupation. (C) Estimates of diagnosis-to-admission distribution stratified by occupation.

In terms of entire cohort, the median time of onset to admission was 8.0 (IQR 5.0-15.0). There were similar probability density distributions for onset-to-diagnosis, onset-to-admission, and diagnosis-to-admission intervals for nurse and doctor patients (Figure 1, A-C).

### Clinical features

There were 19 (19%) cases with one or more co-morbidities. The three most common symptoms were fever (70%), cough (58%) and fatigue (39%). The relatively fewer common symptoms were sore throat, myalgia, difficulty breathing or chest tightness, sputum production, headache, chill, loss of appetite, diarrhea, and chest pain (Table 1).

Table 2 shows the laboratory and radiographic findings of 101 medical staff with confirmed COVID-19. On admission, the blood counts of 23 (23%) cases showed leukocytopenia and only one (1%) showed leukocytosis. 33 (33%) presented with lymphocytopenia and 12 (12%) presented with thrombocytopenia. Most cases demonstrated normal levels of D-dimer, creatinine, and creatine kinase, but elevated C-reactive protein and amyloid A levels were presented in 41% and 59% of cases, respectively. Elevated levels of alanine aminotransferase (15%) and aspartate aminotransferase (8%) were less common. A small proportion (2%) of cases had abnormal procalcitonin serum level (>0.5 ug/L). Notably, 80% of cases had increased levels of IL-6 (>2.9 pg/ml). In contrast to nurses, doctors showed significantly higher levels of creatinine, creatine kinase, lactose dehydrogenase, alanine aminotransferase, aspartate aminotransferase, hemoglobin, ferritin, and total bilirubin (all *P*-values<0.05).

**Figure 2:**
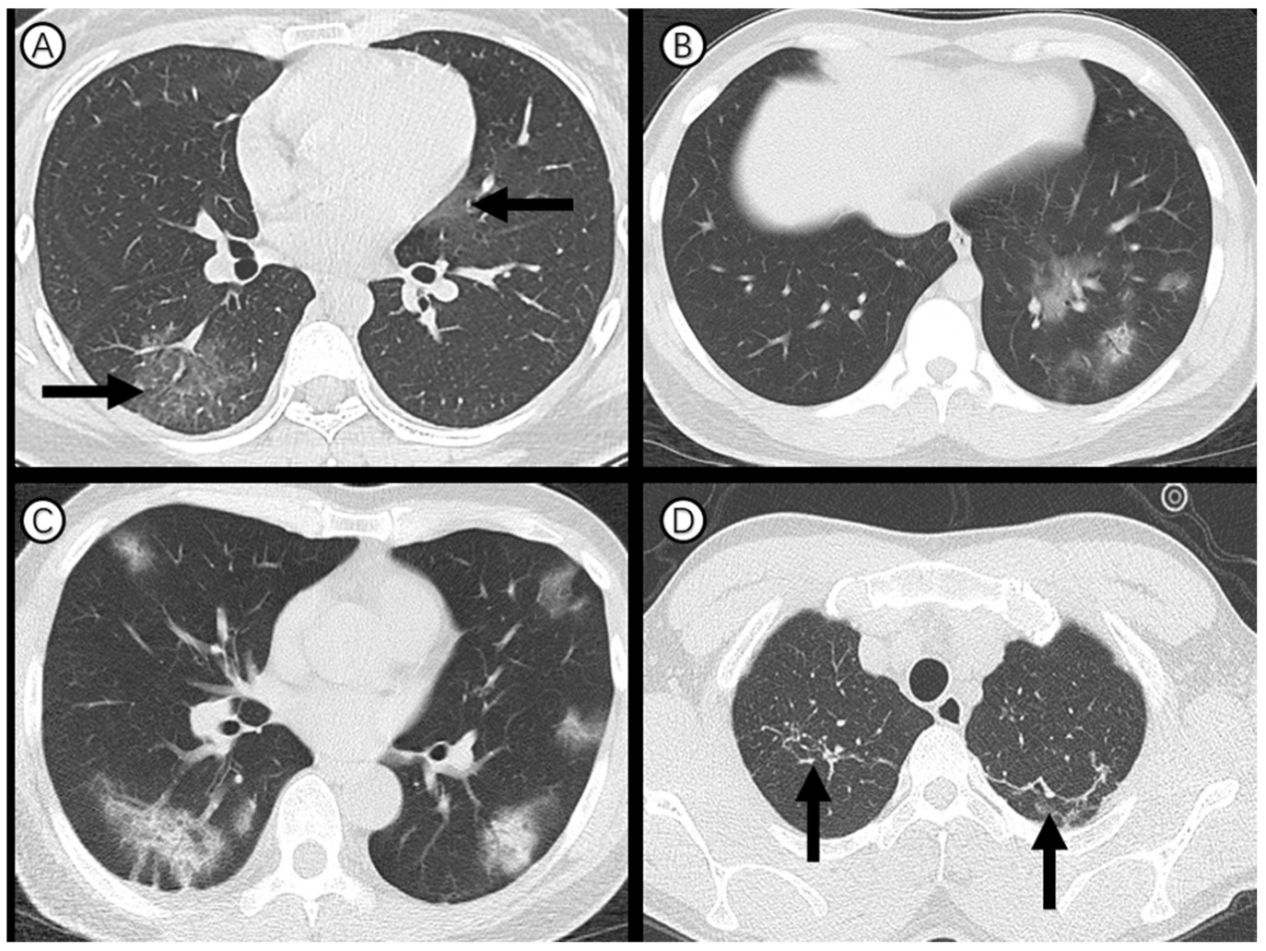
** Axial thin-section CT scans in medical staff infected with SARS-CoV-2.** (A) 27-year-old woman: bilateral, peripheral ground-glass opacity in the right lower lobe and left upper lobe (arrow). (B) 27-year-old man: unilateral, multiple and perbronchovasular ground-glass opacity associated with air bronchograms in the left lower lobe. (C) 55-year-old man: bilateral, peripheral ground-glass opacity mixed consolidation pattern. (D) 28-year-old man: bilateral and linear atelectasis in the right and left upper lobes (arrow), regarded as chronic lung lesion with lack of changes on serial CT examinations.

Ninety-one (90%) of 101 cases showed abnormal chest CT (Figure 2). Sixty-three (62%) had bilateral lung involvement (Figure 2, A and C). The right lower lobe (68 %) and left lower lobe (70%) were the most common involved lobes. Ground glass opacity was the predominant abnormality on chest CT (Figure 2, A and B) and observed in 48 cases (48%). Subpleural distribution was predominant distribution pattern of the ground glass opacity as identified in 78 cases (77%) (Figure 2, C). Adjacent pleura thickening, nodules, emphysema, pleural effusion and lymphadenopathy were relatively rare. CT scans also found that 23 (23%) of medical staff had one or more chronic lung lesions with unchanged appearance on serial CT examinations (Figure 2, D).

### Treatment measures and prognosis

Of the study subjects, no person was transferred to an intensive care unit for mechanical ventilation due to acute respiratory distress syndrome. Empirical intravenous antibiotic treatment was administered in 84 (83%) patients. All the patients were given empirical antiviral therapy. Meanwhile, 37 (37%) were offered traditional Chinese medicine, 34 (34%) patients were given immune globulin, 58 (57%) were given thymosin, and 11 (11%) received corticosteroids. As for oxygen therapy, 56 (55%) used nasal cannula and only 7 (7%) used face mask, while no one needed invasive mechanical or ventilation extracorporeal membrane oxygenation.

**Figure 3:**
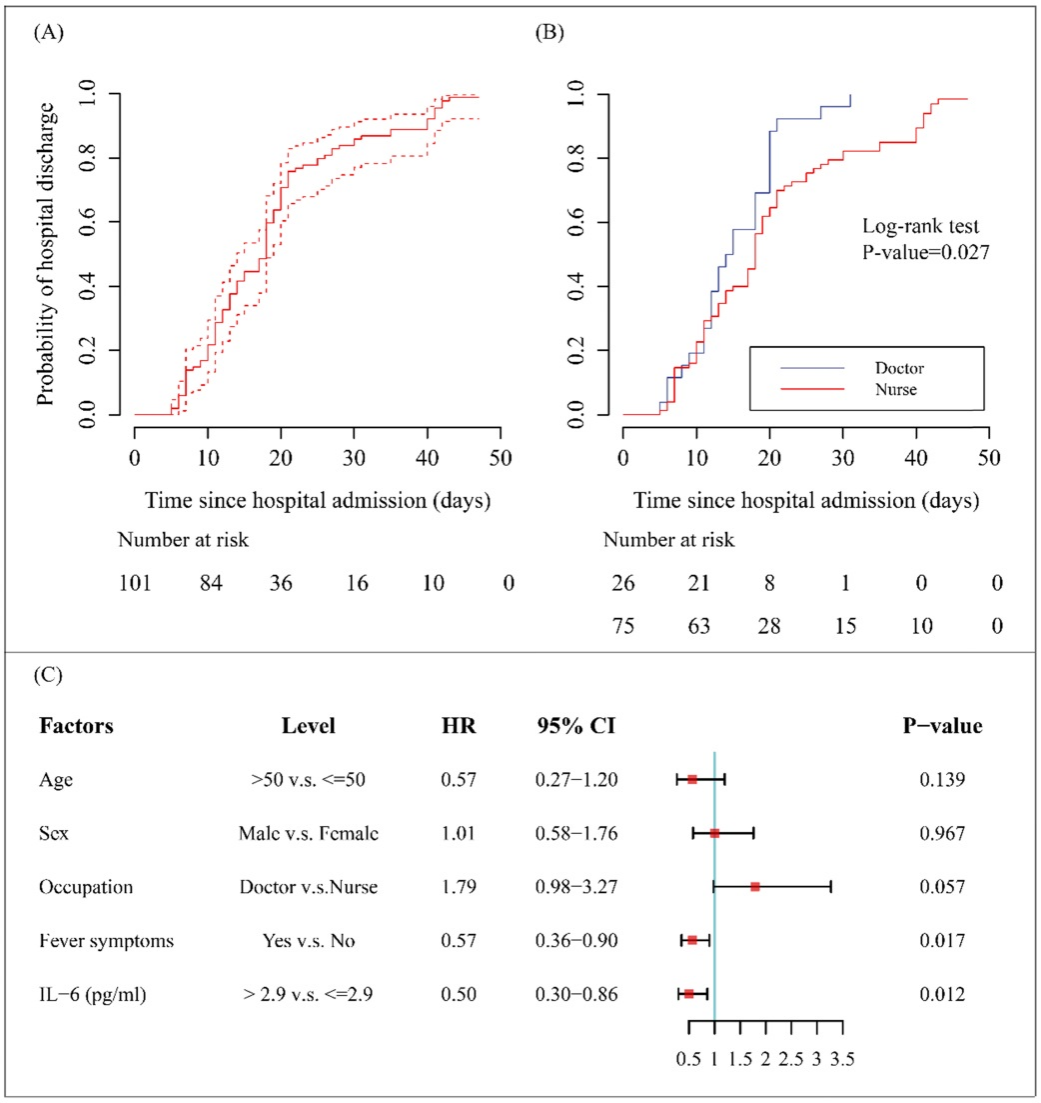
** Hospital discharge rates and factors associated with outcome for medical staff infected with COVID-19 pneumonia.** (A) The probability of hospital discharge and the length of hospitalization for the study subjects. Dotted arrows represent 95% CIs. (B) The probability of hospital discharge and the length of hospitalization stratified by occupation (doctor or nurse). (C) The results of proportional hazard Cox model. Hazard ratios (HRs) and corresponding 95% CIs were displayed for the factors including age, sex, occupation, fever symptoms and IL-6 levels.

By 20 Mar 2020, 98 (97%) of the cases have been discharged and none had died, the remaining 3 cases were still in hospital to receive supportive therapy. According to the results of Kaplan-Meier method, the median discharge time (i.e. equal to the time that half of the patients left the hospital) of the entire cohort was 18.0 (95% CI, 14.0-19.0) days (Figure 3, A). The accumulative probability of hospital discharge was higher in doctors versus nurses examined by log-rank test (*P*-value<0.05) (Figure 3, B).

It should note that the endpoint of final multivariate Cox regression model was discharge, and patients who continued to be hospitalized as of 20 Mar 2020 were regarded as censored data. Fever symptoms (HR=0.57; 95% CI 0.36-0.90) and elevated IL-6 levels (> 2.9 pg/ml) on admission (HR=0.50; 95% CI 0.30-0.86) were unfavorable factors for discharge (all HRs <1 and all *P*-values <0.05) (Figure 3, C). However, the differences in discharge rates between the two occupational types tended to be marginally significant after adjusting for others covariates (*P*-value=0.057) (Figure 3, C).

## Discussion

By 20 Feb, 2020 the China CDC Weekly reported a total of 2,055 laboratory-confirmed cases of medical staff with SARS-CoV-2 infection, of which the majority (88%) were from Hubei province 19. The exact mode of the most medical staff infection (73%) remains unclear in our study, in consistency with the findings reported by Wei X-S et al. 11 and Chu J et al. 13 respectively. Differ from some published studies about ordinary people 8, 9, we found that the infection of SARS-Cov-2 among medical staff mainly occurred at the early stage of COVID-19 epidemic in Wuhan. Possible reasons for these phenomena include lack of knowledge about transmission approaches and experience to fight with the SARS-CoV-2, coupled with a shortage of protection supplies at the early stage 15. Therefore, training the health care professionals on protection techniques and standardized protection process, and providing adequate protective materials may play an important role in preventing infection of the medical staff and facilitating infection control.

The demographic characteristics and clinical manifestations of medical staff with confirmed COVID-19 in Wuhan were not exactly the same as general confirmed patients included in recent studies 10, 20, 21. In our study, most of the novel coronavirus-infected medical staff analyzed were females and nurses, and were younger, in consistency with the findings reported by Xiong W et al 12. The medical staff infected with SARS-CoV-2 have similar signs and symptoms with general confirmed infection patients 10, 21. The infected medical staff tended to have bilateral, subpleural groundglass opacities on chest CT images, which is consistent with the recent radiological reports on COVID-19 pneumonia 22-26. Furthermore, abnormal D-dimer levels as well as abnormal renal, heart and liver function tests were relatively rare among medical staff with SARS-CoV-2 infection.

In our study, only 6 of the 101 medical staffs with SARS-CoV-2 infection were severe case. None developed acute respiratory distress syndrome or transferred to intensive care unit. The low rate of severe and critical case (5.9%, 6/101) of the medical staff infections in our study is similar to the rate of severe cases (5%, 4/80) reported by Wuhan Tongji Hospital, which is a different hospital affiliated to the same university as ours. Previous studies suggested that 13.8% of the general confirmed patients were severe cases, among whom older age, male sex, chronic diseases are more common 27-29. In our study, the medical staffs have relatively milder symptoms, which may be partly due to their medical expertise, younger age and less underlying diseases.

Predictors of hospital discharge among infected medical staff were identified by Cox model. Fever symptoms and elevated IL-6 levels (> 2.9 pg/ml) on admission were significantly associated with lower likelihood of the discharge. Knowledge of how present-on-admission patient factors affect patient's condition and risk during hospitalization is very important because we can use such knowledge to screen and identify patients of higher risk upon admission to the hospital. A recent study revealed that fever was identified in only half of the patients on presentation but increased to nearly 90% after hospitalization 21. Elevated IL-6 levels were observed in 80% of infected medical staff on admission, which is associated with inflammatory response 30, 31. The elevated inflammatory cytokines (such as IL-6 and IL-1) suggest that a cytokine storm may play a major role in the pathology of COVID-19 32.

So far, more than 40,000 health-care workers from 30 provinces gathered in Wuhan for the battle against the epidemic. China has attached great importance to infection prevention among medical staff including providing adequate protective materials (gown, gloves, N95 respirator, face shield or goggles) and training the health care standardized protection process, and the good news was that none of them were infected with SARS-CoV-2 up to now. Meanwhile, some potential problems remain to be solved, such as unclear patterns of infection, mental health care for medical staff 33, and the possibility of airborne transmission from aerosol production by medical practices in health care facilities. A recent study from Singapore found that surface environmental and personal protective equipment contamination caused by respiratory droplets and fecal shedding from patients infected with SARS-CoV-2, suggesting that the environment is a potential viral vector 34. Further investigations should be devoted to identifying the exact patterns of SARS-CoV-2 infection among medical staff.

### Limitations of this study

We acknowledge some limitations of this study. First, only 101 medical staff from a single hospital in Wuhan were included in this study. This limitation may result in deviations in epidemiological and clinical observation characteristics, particularly regarding specific causes of SARS-CoV-2 infection among medical staff; Second, this is a retrospective study and the data used in this study only provide a preliminary insight into epidemiological features and clinical outcomes of a group of medical staff confirmed with COVID-19. Further research on this regard is needed.

## Conclusion

The infection among medical staff mainly occurring at the early stages of COVID-19 epidemic in Wuhan was suggested. In this study, medical staffs infected with COVID-19 have relatively milder symptoms and favorable clinical course than other ordinary patients, which may be partly due to their medical expertise, younger age and less underlying diseases. The major differences between the two occupational types manifested in laboratory indicators. The potential risk factors of presence of fever symptoms and IL-6 levels greater than >2.9 pg/ml could help to identify medical staff with poor prognosis at an early stage. Further investigations should be devoted to identifying the exact mode of COVID-19 among medical staff.

## Figures and Tables

**Table 1:** Demographics and baseline characteristics of medical staff infected with COVID-19 pneumonia in Wuhan, China.

	All (*n*=101)	Occupation		*P*-value
		Doctor (*n*=26)	Nurse (*n*=75)	
Age, median (IQR)	33 (30-41)	37 (31-43)	32 (30-40)	0.124
**Sex**				**<0.001**
Male	32 (32%)	18 (69%)	14 (19%)	-
Female	69 (68%)	8 (31%)	61 (71%)	-
**BMI (kg/m^2^)**	22.0 (20.1-23.4)	22.4 (21.4-24.2)	21.5 (20.0-23.0)	**0.027**
≥ 24	18 (18%)	8 (31%)	10 (13%)	0.071
< 24	83 (82%)	18(69%)	65 (87%)	-
**Current smoking status**				0.273
Yes	4 (4%)	2 (8%)	2 (3%)	-
No	97 (96%)	24 (92%)	73 (97%)	-
**Disease severity**				0.234
Non-severe	95 (94%)	23 (88%)	72 (96%)	-
Severe	6 (6%)	3 (12%)	3 (4%)	-
**Exposure history**				
Exposure to Huanan market	0	0	0	-
Exposure to wildlife	0	0	0	-
Family members as confirmed cases	6 (6%)	2 (8%)	4 (5%)	0.646
Contact with patients in fever clinics	15 (15%)	3 (12%)	12 (16%)	0.754
Contact with patients in isolation wards	3 (3%)	1 (4%)	2 (3%)	1
Contact with specimens	3 (3%)	3 (12%)	0	**0.016**
**Use of preventive medication**				0.276
Yes	10 (10%)	4 (15%)	6 (8%)	-
No	91 (90%)	23 (85%)	69 (92%)	-
**Comorbidities**	19 (19%)	4 (15%)	15 (20%)	0.774
Hypertension	4 (4%)	1 (4%)	3 (4%)	1
Diabetes	1 (1%)	0	1 (1%)	1
Coronary heart disease	1 (1%)	0	1 (1%)	1
Chronic obstructive pulmonary disease	0	0	0	-
Other	14 (14%)	3 (12%)	11 (15%)	1
**Signs and symptoms**				
Fever	71 (70%)	16 (62%)	55 (73%)	0.376
Maximum temperature, °C				0.646
≤37.3	30 (30%)	10 (38%)	20 (27%)	-
37.3-38	42 (42%)	10 (38%)	32 (43%)	-
38-39	20 (20%)	4 (15%)	16 (21%)	-
>39	6 (6%)	2 (8%)	4 (5%)	-
Cough	59 (58%)	15 (58%)	45 (59%)	1
Fatigue	39 (39%)	9 (35%)	30 (40%)	0.801
Sore throat	24 (24%)	7 (27%)	17 (23%)	0.863
Myalgia	18 (18%)	8 (31%)	10 (13%)	**0.071**
Difficulty breathing or chest tightness	21 (21%)	3 (12%)	18 (24%)	0.285
Sputum production	21 (21%)	4 (15%)	17 (23%)	0.611
Headache	14 (14%)	4 (15%)	10 (13%)	0.752
Chill	8 (8%)	2 (8%)	6 (8%)	1
Loss of appetite	8 (8%)	2 (8%)	6 (8%)	1
Diarrhea	11 (11%)	4 (15%)	7 (9%)	0.467
Chest pain	5 (5%)	1 (4%)	4 (5%)	1
Time from symptoms onset to admission, median (IQR) (days)	8.0 (5.0-15.0)	7.0 (5.0-12.5)	8.0 (5.0-15.0)	0.720

There were 26 of 101 novel coronavirus-infected medical staff were doctors and 75 were nurses. Data are presented as medians (interquartile ranges, IQR) and *n* (%). P value of < 0.05 was considered to be statistically significant.

**Table 2:** Laboratory and radiographic findings of medical staff infected with COVID-19 pneumonia in Wuhan, China.

	Normal range	All (*n*=101)	Occupation		*P*-value
			Doctor (*n*=26)	Nurse (*n*=75)	
Leukocytes (×10 ^9^ /L)	3.5-9.5	4.5 (3.6-5.7)	4.8 (3.7-5.6)	4.4 (3.5-5.7)	0.545
Decreased		23 (23%)	4 (15%)	19 (24%)	0.441
Increased		1 (1%)	0	1 (1%)	1
Neutrophilic granulocyte percentage (%)	40-75	56.1 (49.9-62.7)	59.5 (52.0-65.1)	54.4 (49.1-62.4)	0.162
Lymphocytes (×10 ^9^ /L)	1.1-3.2	1.4 (0.9-1.8)	1.3 (0.9-1.9)	1.4 (1.0-1.7)	0.706
Decreased		33 (33%)	12 (46%)	21 (28%)	0.145
Platelets (×10 ^9^ /L)	115-350	190 (144-220)	189 (141-213)	190 (146-228)	0.843
Decreased		12 (12%)	2 (8%)	10 (13%)	0.679
D-dimer (mg/L)^*^	0-0.5	0.2 (0.2, 0.4)	0.3 (0.2, 0.5)	0.2 (0.2, 0.4)	0.226
Creatinine (umol/L)	44-106	65.8 (58.6-77.2)	76.3 (65.4-87.5)	62.6 (57.9-70.7)	**<0.001**
Creatine kinase (U/L)	26-140	54.0 (42.0-88.0)	95.5 (63.8-118)	49.0 (38.0-65.0)	**<0.001**
Lactose dehydrogenase (U/L)	109-245	187 (167-217)	214 (177-274)	185 (164-204)	**0.024**
Alanine aminotransferase (U/L)	5-35	19.0 (13.0-29.0)	26.5 (19.3-49.8)	16.0 (13.0-26.5)	**<0.001**
Increased		15 (15%)	8 (31%)	7 (9%)	**0.020**
Aspartate aminotransferase (U/L)	8-40	22.0 (16.0-26.0)	24.5 (22.0-32.5)	20.0 (16.0-25.0)	**0.002**
Increased		8 (8%)	6 (22%)	2 (3%)	**0.004**
Hemoglobin (g/L)	115-150	128 (119-139)	139 (128-150)	126 (119-134)	**0.002**
Ferritin (ug/L)	4.6-204	99 (55-247)	329 (206-553)	82 (43-155)	**<0.001**
C-reactive protein >8 mg/L	0-8	41 (41%)	13 (50%)	28 (37%)	0.367
Amyloid A (mg/L)	0-10	22.4 (5.0-129.4)	39.6 (7.5-334.3)	15.5 (4.8-78.6)	**0.067**
Increased		50/85 (59%)	18/25 (72%)	32/60 (53%)	0.177
Procalcitonin >0.5 ug/L	0-0.5	2/95 (2%)	1/23 (4%)	1/72 (1%)	0.428
Total bilirubin (umol/L)	5.1-19	8.9 (7.5-12.5)	11.1 (8.7-14.7)	8.5 (67.0-13.4)	**0.003**
IL-6 (pg/ml)	0.1-2.9	4.3 (3.2-6.9)	4.3 (3.3-6.4)	4.3 (3.2-7.0)	0.967
Increased		76/95 (80%)	21/25 (84%)	55/70 (79%)	0.771
lymphocyte subsets					
CD3+ ratio (%)	58.17-84.22	75.2 (70.1-79.9)	70.5 (64.0-76.9)	77.3 (71.9-80.6)	**0.019**
CD4+ ratio (%)	25.34-51.37	41.3 (35.2-46.0)	38.2 (34.2-44.6)	41.7 (36.4-48.4)	0.303
CD8+ ratio (%)	14.23-38.95	27.9 (23.6-32.6)	25.7 (23.6-30.0)	28.4 (24.5-33.4)	0.178
B-CELL ratio (%)	4.1-18.31	11.2 (8.9-14.9)	10.4 (9.2-14.6)	11.3 (8.8-15.3)	0.575
NK cell ratio (%)	3.33-30.47	6.2 (4.0-10.2)	11.1 (6.1-17.7)	5.2 (3.5-7.6)	**0.002**
Ratio of CD4/CD8	0.41-2.72	1.4 (1.2-1.9)	1.5 (1.3-1.9)	1.4 (1.2-1.9)	0.617
Abnormalities on chest CT	-	91 (90%)	25 (96%)	66 (88%)	0.446
Lung involvement					
Unilateral	-	28 (28%)	8 (31%)	20 (27%)	0.882
Bilateral	-	63 (62%)	17 (65%)	46 (61%)	0.895
Lung lobe involved					
Right upper lobe	-	35 (35%)	12 (46%)	23 (31%)	0.234
Right middle lobe	-	29 (29%)	15 (58%)	14 (19%)	**<0.001**
Right lower lobe	-	69 (68%)	20 (77%)	49 (65%)	0.395
Left upper lobe	-	39 (39%)	16 (62%)	23 (31%)	**0.011**
Left lower lobe	-	71 (70%)	17 (65%)	54 (72%)	0.699
Predominant CT pattern					
Ground glass opacity	-	48 (48%)	12 (46%)	36 (48%)	1
Consolidation	-	13 (13%)	4 (15%)	9 (12%)	0.534
Mixed pattern	-	30 (30%)	9 (35%)	21 (28%)	0.699
Predominant distribution of opacities					
Septal/subpleural	-	78 (77%)	21 (81%)	57 (76%)	0.819
Peribronchovascular	-	4 (4%)	0	4 (5%)	0.570
Random	-	9 (9%)	4 (15%)	5 (7%)	0.230
Thickening of the adjacent pleura	-	4 (4%)	2 (8%)	2 (3%)	0.272
Pleural effusion/ Lymphadenopathy		0	0	0	-
Any original chronic lung lesion	-	23 (23%)	7 (27%)	16 (21%)	0.753

Data are presented as medians (interquartile ranges, IQR) and *n* (%). For each item, the effective sample size of total population, group of doctors, group of nurses is 101, 26 and 75.

**Table 3:** Treatments and outcomes of medical staff infected with COVID-19 pneumonia in Wuhan, China.

	All (*n*=101)	Occupation		*P*-value
		Doctor (*n*=26)	Nurse (*n*=75)	
Electrocardiograph monitoring	24 (24%)	6 (23%)	18 (24%)	1
Antibiotics treatment	84 (83%)	19 (73%)	65 (87%)	0.132
Antiviral treatment	101 (100%)	26 (100%)	75 (100%)	-
Traditional Chinese medicine	37 (35%)	7 (26%)	30 (38%)	0.401
Immune globulin	34 (34%)	9 (35%)	25 (33%)	1
Thymosin	58 (57%)	14 (54%)	44 (59%)	0.843
Corticosteroids	11 (11%)	2 (8%)	9 (12%)	0.724
Oxygen therapy				
Nasal cannula	56 (55%)	13 (50%)	43 (57%)	0.675
Face mask	7 (7%)	3 (12%)	4 (5%)	0.370
Length of hospital stay (days)	17.0 (11.0-21.0)	14.0 (11.3-20.0)	18.0 (11.0-23.0)	0.232
Outcome				0.567
Hospital discharge	98 (97%)	26 (100%)	72 (96%)	-
Continued hospitalization	3 (3%)	0	3 (4%)	-
Death	0	0	0	-

There were 26 of 101 novel coronavirus-infected medical staff were doctors and 75 were nurses. Data are presented as medians (interquartile ranges, IQR) and *n* (%).
